# Differential Expression of AP-2 Transcription Factors Family in Lung Adenocarcinoma and Lung Squamous Cell Carcinoma—A Bioinformatics Study

**DOI:** 10.3390/cells12040667

**Published:** 2023-02-20

**Authors:** Dagmara Szmajda-Krygier, Adrian Krygier, Marta Żebrowska-Nawrocka, Jacek Pietrzak, Rafał Świechowski, Agnieszka Wosiak, Agnieszka Jeleń, Ewa Balcerczak

**Affiliations:** Laboratory of Molecular Diagnostics and Pharmacogenomics, Department of Pharmaceutical Biochemistry and Molecular Diagnostics, Medical University of Lodz, 90-151 Lodz, Poland

**Keywords:** AP-2, *TFAP2* genes, lung cancer, NSCLC, LUAD, LUSC, transcription factors

## Abstract

Members of the activator protein 2 (AP-2) transcription factor (TF) family are known to play a role in both physiological processes and cancer development. The family comprises five DNA-binding proteins encoded by the *TFAP2A* to *TFAP2E* genes. Numerous scientific reports describe differential expression of these TF and their genes in various types of cancer, identifying among them a potential oncogene or suppressor like *TFAP2A* or *TFAP2C*. Other reports suggest their influence on disease development and progression, as well as response to treatment. Not all members of this AP-2 family have been comprehensively studied thus far. The aim of the present article is to gather and discuss knowledge available in bioinformatics databases regarding all five members of this family and to differentiate them in relation to the two most common lung cancer subtypes: adenocarcinoma (LUAD) and squamous cell carcinoma (LUSC). In addition, to assess the difference in levels depending on a number of clinicopathological factors, the impact on patient survival and interactions with tumor-infiltrating immune cells. This article may help to identify the target for further original research that may contribute to the discovery of new diagnostic biomarkers and define the molecular differences between LUAD and LUSC, which may affect the therapy effectiveness improvement and longer survival.

## 1. Introduction

Lung cancer is the most commonly diagnosed malignancy and the leading cause of cancer-related mortality worldwide. Non-small cell lung cancers (NSCLC) comprise up to 85% of all lung tumors and are divided according to histological subtypes to lung adenocarcinoma (LUAD) and lung squamous cell carcinoma (LUSC) [1,2]. Studies of lung cancer genome have shown that several genes are likely to be key mediators of tumor initiation and progression, including genes such as *EGFR*, *FGFR1* and *2*, *ALK*, *RET*, *KRAS*, *NOTCH1*, *TP53*, *SOX* and many more, together with activating enhancer-binding protein 2 (AP-2) and the transcription factor (TF) gene family (*TFAP2*). However, experimental validation of the most important functional genomic alterations in lung cancer cells remains challenging [2,3]. It also remains an important task to understand which of the wide variety of alterations are significant in lung carcinogenicity and/or treatment response, as opposed to those changes that are only a consequence of the neoplastic process. The profiling of genomic alterations in cancer highlights the heterogeneity of the NSCLC patients’ genomes and provides a reliable explanation for differences observed clinically and in individual response to therapy. The creation of specific mutation catalogs for each patient will help to obtain a more accurate assessment of all potential effects of the genotype and treatment response, ultimately leading to the selection of an appropriate treatment strategy tailored to the patient [1,3]. It is well known that both, the pathological characteristics and treatment response of NSCLC patients are influenced by genetic and epigenetic mechanisms, as well as the origin of the tumor’s cells [2]. What is more, the latest research suggests that LUAD and LUSC differ at the molecular, pathological, and clinical levels and should be classified and treated as distinct entities [1,3]. Identification of the mechanisms underlying LUAD and LUSC pathogenesis is necessary to develop biomarkers and tools for better diagnosis and successful therapy. Multigene expression and immunohistochemistry analyses helped to identify some biological pathways and biomarkers that differentiate LUAD and LUSC subtypes [1,4,5].

One of them seems to be the *TFAP2* gene family, and in the present work information about the molecular role of these genes in subtypes of LUAD and LUSC are gathered. The AP-2 family of transcription factors in humans consists of five members: AP-2alfa, AP-2beta, AP-2gamma, AP-2delta, and AP-2epsilon (AP-2α, AP-2β, AP-2γ, AP-2δ, and AP-2ε). The proteins can form hetero- as well as homodimers [6,7]. AP-2 proteins present in structure a conserved motif of helix-span-helix dimerization at the C terminus, followed by a central basic region and a less conserved domain with proline and glutamine at the N terminus. The helix-span-helix motif with the basic region is responsible for DNA binding, whereas the proline and glutamine-rich region is necessary for transactivation [8,9]. AP-2 members are able to bind to those commonly present in different organisms’ palindromic sequences, like 5′-GCCN3GGC-3′, 5′-GCCN4GGC-3′ and 5′-GCCN3/4GGG-3′ found in various cellular enhancers, which confirms their crucial roles [10]. AP-2 proteins can also bind with variable affinity to a range of G/C-rich elements. The genes with AP-2-binding sites in their promoter sequences are also involved in biological processes, such as cell growth and differentiation and include many receptors, growth factors and more [8]. In the transactivation domain, the majority of AP-2 proteins have PY motif (xPPPxY sequence) and other highly conserved critical residues. This motif is missing in AP-2δ but other domains, mentioned above, are still conserved. As a consequence of that modification, the binding affinity of AP-2δ to sites in promoters of regulated genes is lower than that of other AP-2 proteins, which suggests that AP-2δ might transactivate genes by different mechanism [11,12]. The activity of AP-2 proteins is controlled at multiple levels by checking their transactivation potential, DNA binding, subcellular localization, and degradation. Mechanisms of regulation are connected with posttranslational modifications, such as phosphorylation, sumoylation, and redox regulation [6,8]. Moreover, other interacting proteins (APC, PARP, P53, c-Myc, and many more) can modulate the activity or function of AP-2 proteins by binding to them [7,8]. AP-2 transcription factors are localized mostly in the nucleus, where they bind to target sequences and regulate transcription of target genes. These TFs can also interact with other signal transduction pathways, like the downstream pathway of the developmental signaling molecule Wnt by associating with the APC protein in the nucleus [13]. AP-2 proteins not only regulate gene expression directly by binding to the regulatory regions of some of crucial genes but also indirectly, displaying functional protein–protein interactions with other TFs such as c-Myc, pRB, and P53 [6,10]. The loss of AP-2 transcription factor activity leads to proliferation and induces premature differentiation and/or apoptosis in various cell types during development. AP-2 proteins can be called as gatekeepers controlling the balance between proliferation and differentiation during embryogenesis [7]. The function of AP-2 in neoplasms is dual. They act as inhibitors or promoters, which depends on the organs, tissue, clinical stages, histological grades and difference between five family members [12,14]. The *TFAP2A* and *TFAP2C* genes were reported to be involved in tumorigenesis as tumor suppressors in melanomas [15,16], breast [17,18], gastric [19], prostate [20], colorectal [13], and lung cancer [21]. The oncogenic function of AP-2α and AP-2γ was found in neuroblastoma [22], melanomas [23], pancreatic [24], breast [25], and lung cancer [26,27].

The data about the role of AP-2 transcription factors family members in NSCLC, and further, in the most frequent subtypes—LUAD and LUSC—is scarce. Therefore, the aim of the present article is to gather and discuss knowledge available in bioinformatics databases regarding all five members of this family and to differentiate them in relation to the two most common NSCLC subtypes: LUAD and LUSC. In addition, to assess the difference in levels depending on a number of clinicopathological factors, the impact on patient survival and interactions with tumor-infiltrating immune cells. This article may help to identify the target for further original research that may contribute to the discovery of new diagnostic biomarkers and define the molecular differences between LUAD and LUSC, which may affect the therapy effectiveness improvement and longer survival.

## 2. Materials and Methods

### 2.1. The mRNA Expression of Five TFAP2 Family Members in Cancer versus Normal Tissues

The AP-2 transcription factors family genes expression in cancer versus normal tissue were evaluated by using the Tumor Immune Estimation Resource (TIMER) (https://cistrome.shinyapps.io/timer/, accessed on 10 November 2022) [28] and the Gene Expression Profiling Interactive Analysis (GEPIA) (http://gepia.cancer-pku.cn/index.html, accessed on 14 November 2022) [29]. The DiffExp module was chosen in the TIMER tool, which allows for evaluating the differential expression between tumor and adjacent normal tissues for five *TFAP2* gene family members. The Cancer Genome Atlas (TCGA) RNA-seq database was used, data is presented as box plots, and statistical significance was evaluated by the Wilcoxon test. In the GEPIA database, the box plots were generated by using the Expression DIY tool with the LUAD and LUSC datasets applying TCGA normal and GTEx data. The p-values were calculated by one-way ANOVA, using disease state (tumor or normal) as variable for calculating differential expression.

### 2.2. TFAP2 Family Members Expression Analysis Based on Clinical Characteristics in LUAD and LUSC

The UALCAN web tool with default settings (http://ualcan.path.uab.edu/index.html, accessed on 16 November 2022) [30,31] was applied to generate individual expression box plots of *TFAP2A-TFAP2E* genes’ expression levels according to chosen clinical factors. The chosen factors comprised individual cancer stage (stage 1–stage 4), race (Caucasian, African American, Asian), gender (male, female), age (21–40 yrs., 41–60 yrs., 61–80 yrs., 81–100 yrs.), smoking habits (nonsmoker, smoker, reformed smoker (<15 years), reformed smoker (>15 years)) and *TP53* status (mutant, nonmutant). The data is presented as individual box plots and statistical significance was evaluated by Student’s *t*-test.

### 2.3. Immune Cells Infiltration Analysis Related to TFAP2 Family Members Expression in LUAD and LUSC

The TIMER database was also used to evaluate the specific correlations between *TFAP2* gene family members with immune cells in LUAD and LUSC (https://cistrome.shinyapps.io/timer/, accessed on 10 November 2022) [28]. Infiltrating immune cells included by TIMER algorithm are CD4+T cells, CD8+T cells, B cells, neutrophils, macrophages, and dendritic cells. The data is displayed as scatterplots presenting the purity-corrected partial Spearman’s rho value and statistical significance.

### 2.4. Survival Analysis Related to TFAP2 Family Members Expression in LUAD and LUSC

The Kaplan–Meier plotter (https://kmplot.com/analysis/, accessed on 20 November 2022) [32] was used to compare overall survival (OS) between two groups split by individual expression level of five *TFAP2* gene members (low vs. high expression level) in lung cancer, LUAD, and LUSC subtypes. OS was estimated with the follow-up threshold of 60 months (5 years) based on the “autoselect best cutoff” option, excluding biased arrays. Individual p-values were calculated by the log–rank test between the two groups.

### 2.5. TFAP2 Family Members Mutations, Structural Variants and Copy Number Alterations in LUAD and LUSC

Mutations frequencies and the summary of the gene types in LUAD and LUSC tissues were evaluated according to the online instructions of cBioPortal (http://www.cbioportal.org/, accessed on 20 November 2022) [33,34]. The gene mutation status was analyzed by using the tool of cBioPortal based on TCGA database.

In all aforementioned analyses, a *p*-value < 0.05 was regarded as statistically significant.

## 3. Results

### 3.1. The mRNA Expression of Five TFAP2 Family Members in Cancer versus Normal Tissues and in LUAD and LUSC Subtypes

**Figure 1:**
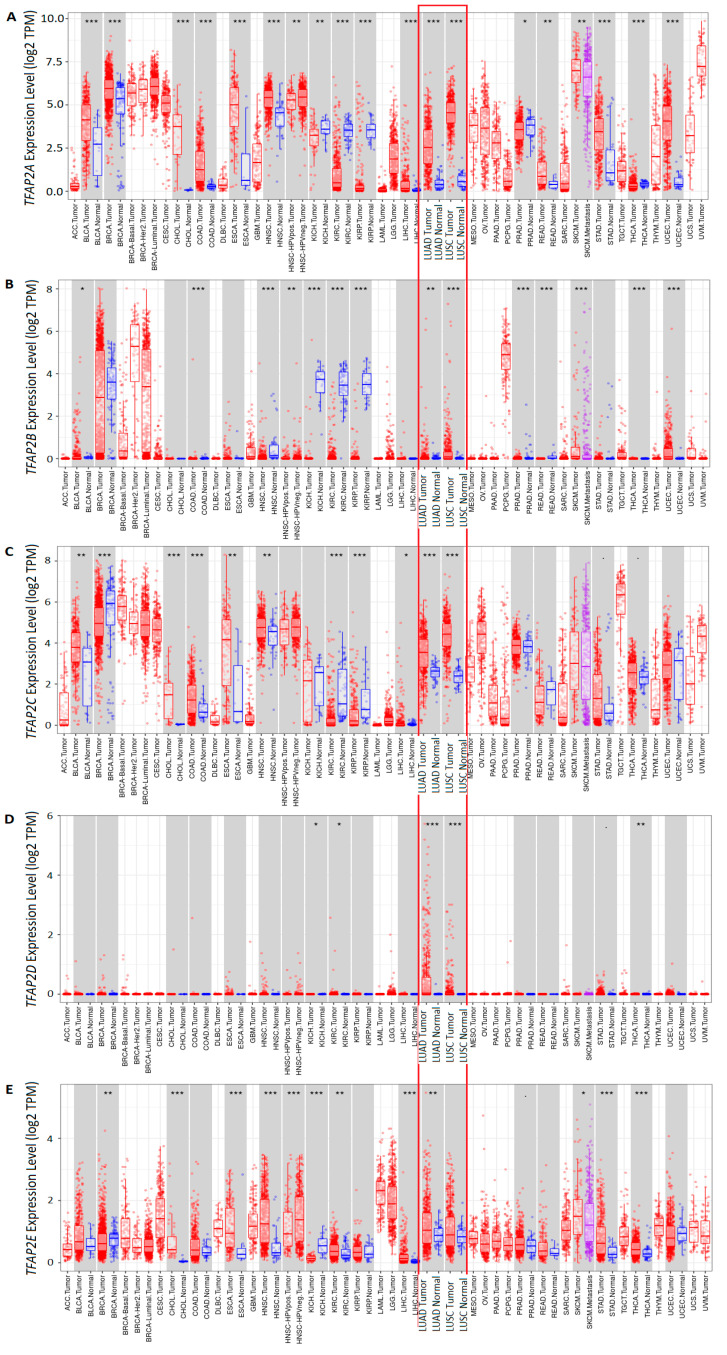
The mRNA expression of *TFAP2* family genes in various cancer types and corresponding normal tissues from the Tumor Immune Estimation Resource (TIMER) database (https://cistrome.shinyapps.io/timer/, accessed on 10 November 2022) [28]. Data presented as red (tumor) and blue (normal tissue) box plots for (**A**) *TFAP2A*, (**B**) *TFAP2B*, (**C**) *TFAP2C*, (**D**) *TFAP2D*, and (**E**) *TFAP2E*. The dots represent the raw data. Significant differences are shown with asterisks: * *p* < 0.05, ** *p* < 0.01, *** *p* < 0.001. The red frame denotes lung adenocarcinoma (LUAD) and squamous cell carcinoma (LUSC) data.

The mRNA expression of *TFAP2* gene family members was identified in various cancer types and normal adjacent tissue pairs using the TIMER database. In particular, the overexpression of *TFAP2A* (Figure 1A) was mostly observed among cancers, including LUAD and LUSC (*p* < 0.01), whereas the downregulation of this gene was only observed in kidney chromophobe (KICH), kidney renal clear cell carcinoma (KIRC), kidney renal papillary cell carcinoma (KIRP) and prostate adenocarcinoma (PRAD). The *TFAP2B* gene expression was mostly found in normal adjacent tissues obtained from KICH, KIRC, and KIRP patients (Figure 1B), whereas this gene was severely underexpressed in paired tumor samples. Nevertheless, in LUAD and LUSC, this gene was found to be overexpressed compared to normal tissue (*p* < 0.001, Figure 1B). Similar to *TFAP2A*, the *TFAP2C* gene was mainly overexpressed among various cancers (Figure 1C) and this observation is true for both LUAD and LUSC subtypes (*p* < 0.001). The downregulation in *TFAP2C* was observed in breast-invasive carcinoma (BRCA), KIRC, and KIRP, compared to normal cells. For most tissues, the expression of *TFAP2D* gene was very low or there was none in most cancer types, and the differences between normal and cancer cells were not significant, although for LUAD and LUSC types the overexpression of this gene was found (Figure 1D, *p* < 0.001). Similar to other family members, the overexpression of *TFAP2E* in comparison to normal tissue was rather characteristic for cancers, including LUAD (*p* < 0.01) but, surprisingly, not LUSC (*p* > 0.05). The downregulation was found in BRCA and KICH tumor samples (Figure 1E).

**Figure 2:**
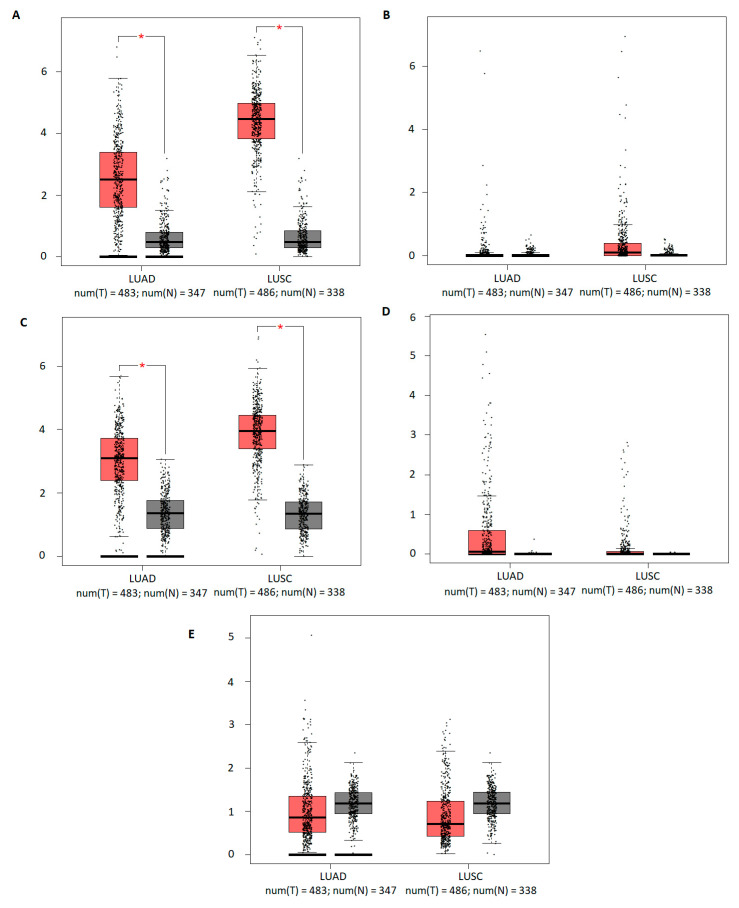
The mRNA expression of *TFAP2* family members in lung adenocarcinoma (LUAD) and squamous cell carcinoma (LUSC) via the Gene Expression Profiling Interactive Analysis (GEPIA) database (http://gepia.cancer-pku.cn/index.html, accessed on 14 November 2022) [29]. Data presented as red (tumor) and gray (normal tissue) box-plots for (**A**) *TFAP2A*, (**B**) *TFAP2B*, (**C**) *TFAP2C*, (**D**) *TFAP2D*, and (**E**) *TFAP2E*. The black dots represent the raw data. Significant differences are shown with asterisks: * *p* < 0.05; T, tumor; N, normal.

In contrast to the presented above results, after examining data for the LUAD and LUSC subtypes in the GEPIA database, the significant differences between tumor and adjacent normal tissues were obtained only for *TFAP2A* and *TFAP2C* genes (*p* < 0.05), where the overexpression for both subtypes were shown (Figure 2). The observed dissimilarities between databases may result from the use of a diverse statistical tests (Wilcoxon test in TIMER and one-way ANOVA in GEPIA database) and a different number of samples analyzed (TCGA RNA-seq dataset in TIMER and TCGA normal and additionally GTEx data in case of GEPIA database).

### 3.2. The Results of TFAP2 Gene Family Members Expression Analysis Based on Clinical Characteristics in LUAD and LUSC

**Figure 3:**
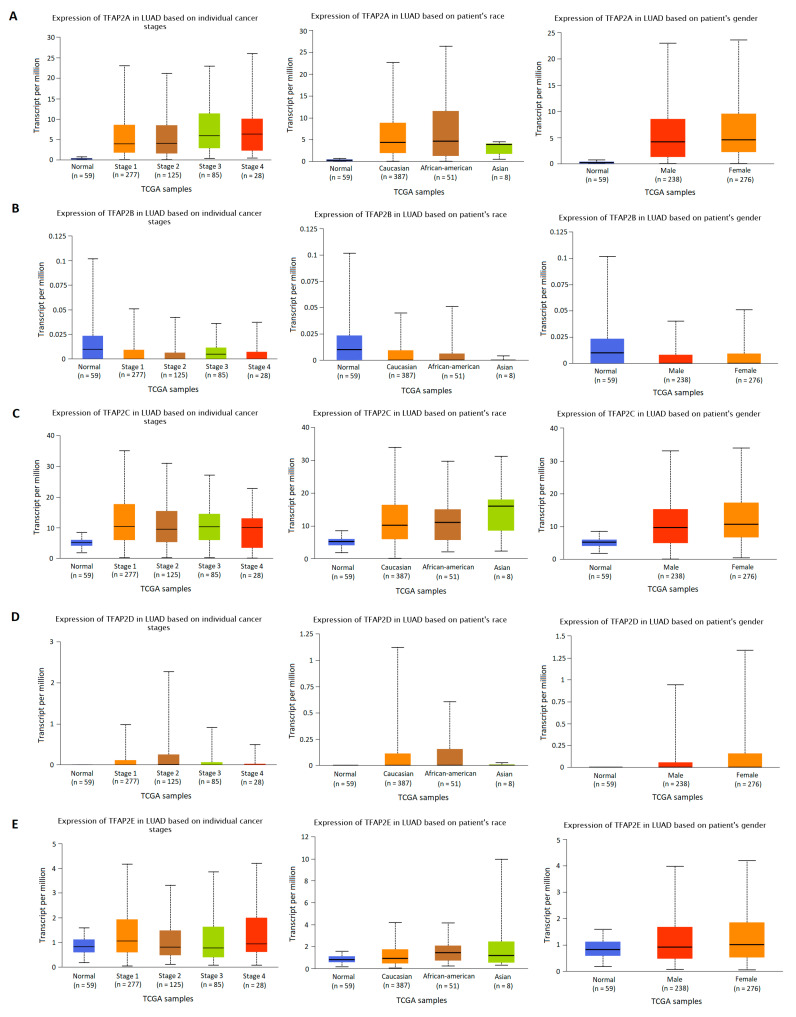
Differences in *TFAP2* gene family members expression according to the clinicopathological factors of lung adenocarcinoma (LUAD) patients. UALCAN web tool with the default settings (http://ualcan.path.uab.edu/index.html, accessed on 16 November 2022) [30,31] was applied to generate individual expression boxplots of *TFAP2A* (**A**), *TFAP2B* (**B**), *TFAP2C* (**C**), *TFAP2D* (**D**), and *TFAP2E* (**E**) genes expression levels according to individual cancer stage, race and gender.

**Figure 4:**
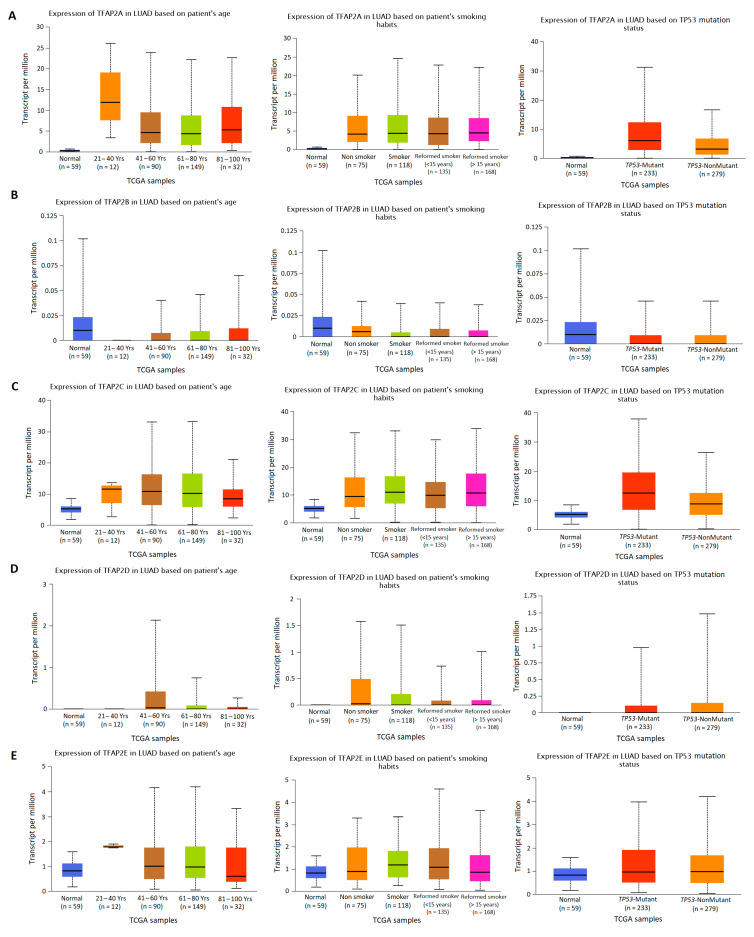
Differences in *TFAP2* gene family members expression according to the clinicopathological factors of lung adenocarcinoma (LUAD) patients. UALCAN web tool with the default settings (http://ualcan.path.uab.edu/index.html, accessed on 16 November 2022) [30,31] was applied to generate individual expression box plots of *TFAP2A* (**A**), *TFAP2B* (**B**), *TFAP2C* (**C**), *TFAP2D* (**D**), and *TFAP2E* (**E**) gene expression levels according to individual age, smoking habits, and *TP53* status.

**Figure 5:**
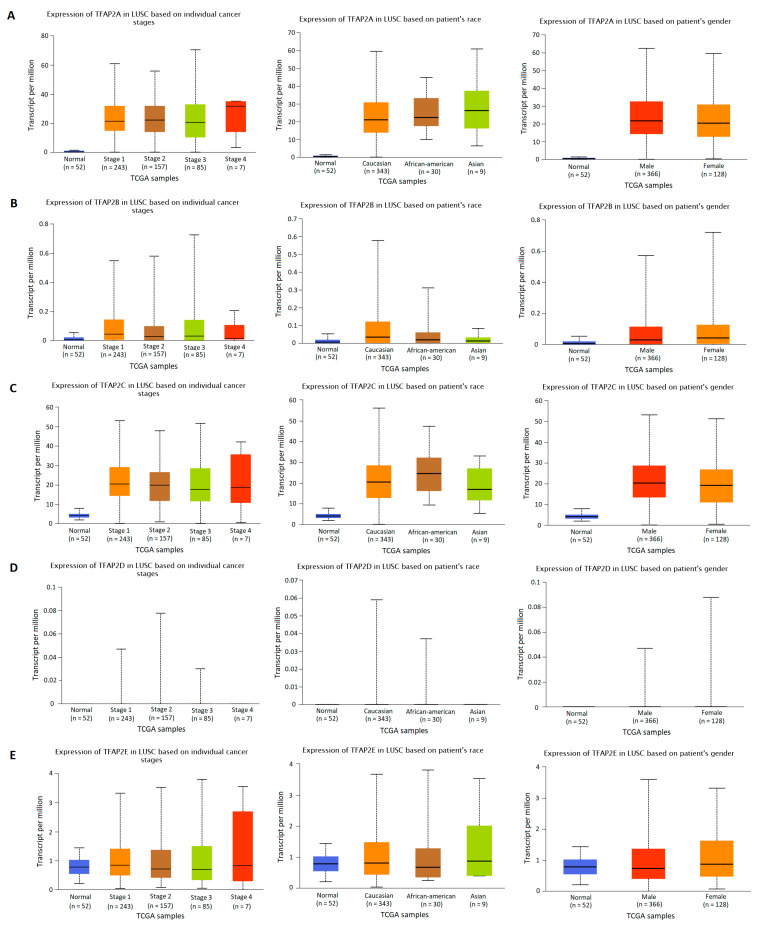
Differences in *TFAP2* gene family members expression according to the clinicopathological factors of lung squamous cell carcinoma (LUSC) patients. UALCAN web tool with the default settings (http://ualcan.path.uab.edu/index.html, accessed on 16 November 2022) [30,31] was utilized to generate individual expression boxplots of *TFAP2A* (**A**), *TFAP2B* (**B**), *TFAP2C* (**C**), *TFAP2D* (**D**), and *TFAP2E* (**E**) gene expression levels according to individual cancer stage, race, and gender.

**Figure 6:**
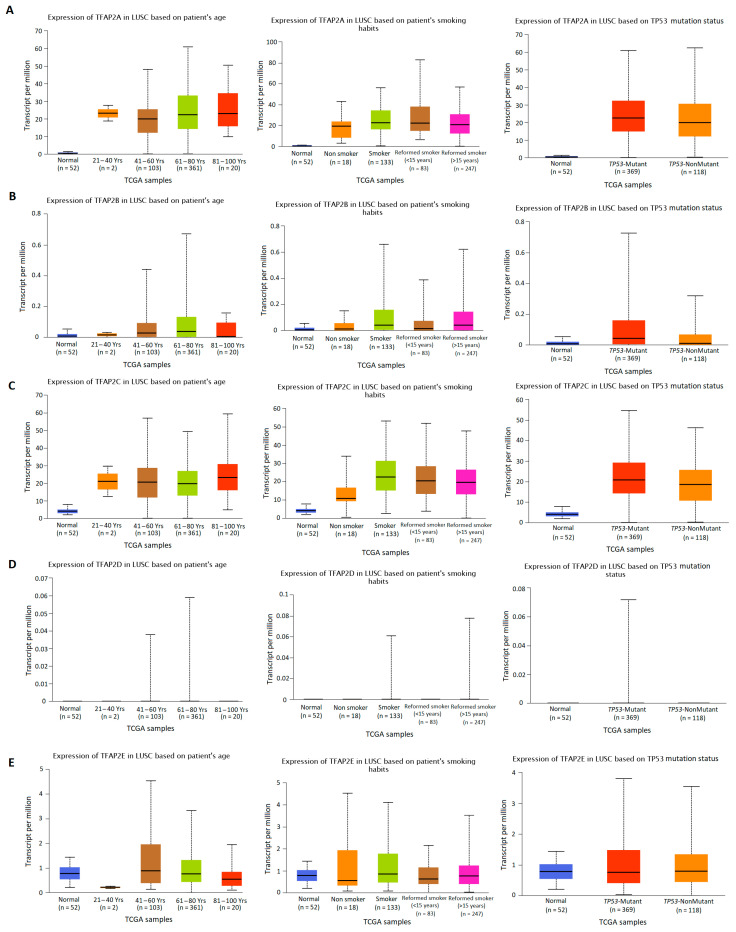
Differences in *TFAP2* gene family members expression according to the clinicopathological factors of lung squamous cell carcinoma (LUSC) patients. UALCAN web tool with the default settings (http://ualcan.path.uab.edu/index.html, accessed on 16 November 2022) [30,31] was utilized to generate individual expression boxplots of *TFAP2A* (**A**), *TFAP2B* (**B**), *TFAP2C* (**C**), *TFAP2D* (**D**), and *TFAP2E* (**E**) gene expression levels according to individual age, smoking habits and *TP53* status.

The *TFAP2* gene family mRNA expression was analyzed according to numerous clinicopathological factors in LUAD (Figure 3 and Figure 4) and LUSC (Figure 5 and Figure 6) patients by using the UALCAN analysis tools. The chosen factors comprised individual cancer stage, race, gender, age, smoking habits, and *TP53* status. The analysis of the *TFAP2A* gene expression level in relation to the abovementioned clinical and demographic characteristics among adenocarcinoma patients (Figure 4A) showed a statistically significant difference only in the context of *TP53* status (*p* < 0.001). Higher levels of expression were observed in mutant *TP53* LUAD patients compared to wild-type *TP53* patients. No significant differences in *TFAP2B* gene expression levels were noticed depending on LUAD patients’ tumor stage, race, gender, age, smoking status, and presence of mutations in the *TP53* gene (Figure 3B and Figure 4B). This may be due to the fact that the TPM values for gene considered are extremely low (TPM < 1). Similar to *TFAP2A* gene, the only significant analysis concerning the *TFAP2C* gene expression level (Figure 4C) showed a difference in relation to the *TP53* status (*p* < 0.001). Higher levels of expression were seen in mutant *TP53* patients compared to wild-type *TP53* patients. The analysis of *TFAP2D* gene in LUAD subtype (Figure 3D) revealed that significantly lower expression level was observed at stage 4 of LUAD vs. stage 1 (*p* < 0.001), stage 2 (*p* = 0.0063) and stage 3 (*p* = 0.0068), which may indicate inhibition of *TFAP2D* gene expression along with tumor development. No other associations for *TFAP2D* gene were found. In case of *TFAP2E* analysis (Figure 3E) it was shown that a significantly higher gene expression level was observed at stage 1 of LUAD vs. stage 2 (*p* = 0.0049) and stage 3 (*p* = 0.0294), which may indicate that activation of this gene is characteristic in early tumor development. Furthermore, analysis for *TFAP2E* gene showed a difference in relation to the *TP53* status (*p* = 0.0223, Figure 4E). Higher levels of expression were observed in mutant *TP53* patients compared to wild-type *TP53* patients.

Slightly different results were obtained in the group of patients with LUSC (Figure 5 and Figure 6). The analysis of the *TFAP2A* gene expression level in relation to the clinicopathological characteristics of squamous cell carcinoma patients showed a statistically significant correlation only with regard to smoking status (Figure 6A). Lower levels of *TFAP2A* gene expression were observed in patients who did not smoke for at least 15 years compared to active smokers (*p* = 0.021). Significantly higher *TFAP2A* transcript level was also found in patients who did not smoke for less than 15 years compared to those who quit smoking at least 15 years earlier (*p* = 0.0095). However, there was no difference in the expression of the *TFAP2A* gene between smokers and nonsmokers, which may be the result of the small number of nonsmokers included in the study (*n* = 18). The analysis of *TFAP2B* gene in LUSC subtype (Figure 5B) revealed that significantly lower expression level was observed at stage 4 of lung cancer vs. stage 1 (*p* = 0.0423), which may indicate inhibition of *TFAP2B* gene expression during tumor development. Higher expression level of this gene was also observed in Caucasians compared to Asians (*p* = 0.0257) and Afro-Americans (*p* = 0.0344). Moreover, *TFAP2B* gene expression was higher in patients diagnosed between 61–80 years of age compared to both younger (41–60; *p* = 0.0219) and older (81–100; *p* = 0.019) patients (Figure 6B). Smoking status is the last parameter for which a statistically significant relationship was demonstrated (Figure 6B). Smokers had higher expression of *TFAP2B* gene than nonsmokers (*p* = 0.0133), but compared to reformed smokers, these differences were no longer significant. For the other analyzed parameters, no statistically significant differences were found. The results of analysis performed for *TFAP2C* gene (Figure 5C and Figure 6C) indicate the significant difference in gene expression level according to smoking status of LUSC patients, the levels were lower in nonsmokers as compared to active smokers (*p* = 0.0245) and reformed smokers for less than 15 years (*p* = 0.0103). What is more, the significance is lost when the results are compared to the reformed smokers, who quit tobacco over 15 years earlier. This may indicate that active smoking can induce abnormal activation of this gene, the effects of which can last up to 15 years. Moreover, a difference in relation to the *TP53* status and *TFAP2C* gene expression level was found (*p* = 0.0079). Higher levels of expression were seen in mutant *TP53* patients compared to wild-type *TP53* patients. The expression levels of the *TFAP2D* gene in LUSC were below the detection limits (Figure 5D and Figure 6D) and no significant differences were found in all aforementioned clinical factors (*p* value not available for most cases). In case of *TFAP2E* gene expression level analysis (Figure 5E and Figure 6E), the only significant difference was observed between smokers and reformed smokers of less than 15 years (0.0082), and slight difference between reformed smokers of less than 15 years, and those who quit tobacco over 15 years earlier (*p* = 0.0454). The levels were higher in smokers and in those, who quit smoking over 15 years earlier. No other associations for this gene were found.

### 3.3. The Correlation between TFAP2 Gene Family Members Expression and Immune Cells Infiltration in LUAD and LUSC

**Figure 7:**
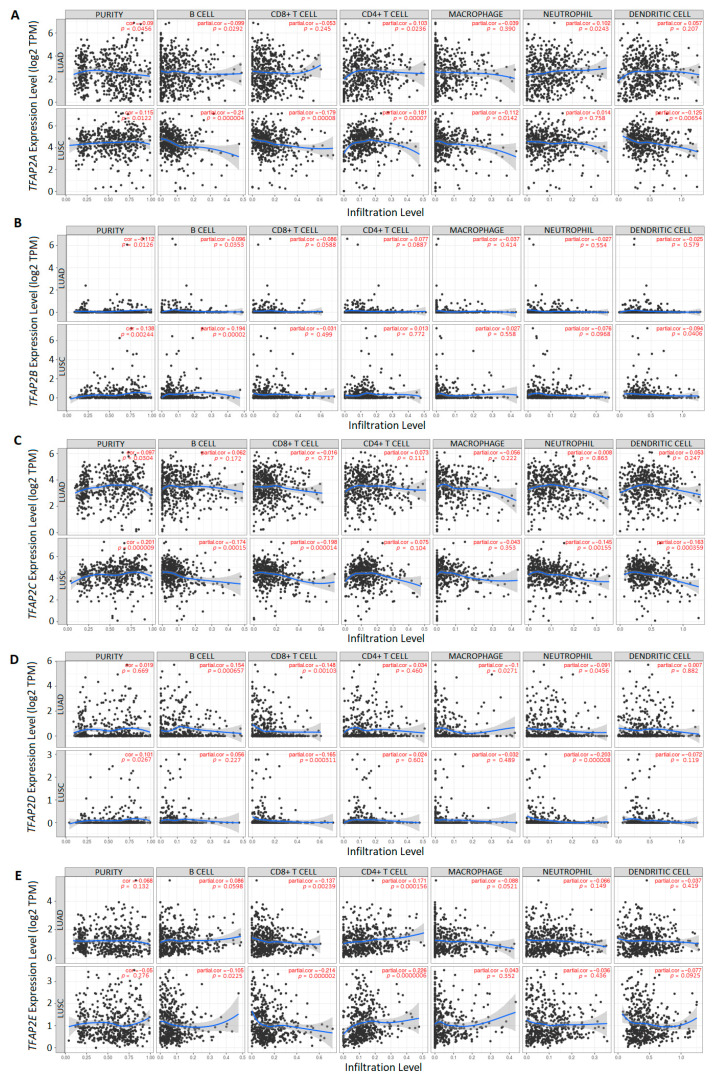
The correlation between *TFAP2* family genes and immune cell infiltration via the Tumor Immune Estimation Resource (TIMER) database (https://cistrome.shinyapps.io/timer/, accessed on 10 November 2022) [28]. The correlation between the abundance of immune cell and the expression of (**A**) *TFAP2A*, (**B**) *TFAP2B*, (**C**) *TFAP2C*, (**D**) *TFAP2D*, and (**E**) *TFAP2E* in lung adenocarcinoma (LUAD) (upper sections) and lung squamous cell carcinoma (LUSC) (lower sections). Black dots represent raw data, the blue line represents partial correlation.

In the next step, we evaluated the association between *TFAP2* gene family members expression and LUAD and LUSC immune cell infiltration by using the TIMER database. The results of analyzing the *TFAP2A* gene (Figure 7A) showed that the gene expression was negatively correlated to LUAD tumor purity, which means that the cells of the neoplastic microenvironment are characterized by a slightly higher expression of this gene in relation to the LUAD cells (*p* = 0.0456). In the case of LUSC, the situation is opposite and higher expression of the *TFAP2A* gene correlates with cancer cells (*p* = 0.012). Furthermore, the expression of this gene is correlated negatively for LUAD and LUSC with B cells (*p* = 0.0292 and *p* < 0.001) and positively with CD4+ T cells (*p* = 0.0236 and *p* < 0.001). Additionally, the significant positive correlation was also observed for neutrophils (*p* = 0.0243) in LUAD and negative for CD8+ T cells (*p* < 0.001), macrophages (*p* = 0.0142), and dendritic cells (*p* = 0.0065) in LUSC. The analysis of the *TFAP2B* gene expression level (Figure 7B) showed the negative correlation with tumor purity in patients with LUAD (*p* = 0.0126) and conversely, the positive correlation in LUSC tissues (*p*= 0.0024). This indicates that cancer microenvironment cells are characterized by a slightly higher expression of the *TFAP2B* gene in relation to LUAD cells and the situation is reversed in LUSC, in which higher expression of the *TFAP2B* gene is typical for cancer cells. The tumor-infiltration immune cells, in which the expression of the *TFAP2B* gene correlates positively, are B lymphocytes in both subtypes of lung cancer (LUAD *p* = 0.0353, LUSC *p* < 0.001) and, additionally, negatively with dendritic cells in the case of squamous cell carcinoma (*p* = 0.0406). Expression level of *TFAP2C* mRNA (Figure 7C) was positively correlated with tumor purity for both types of lung cancer—LUAD (*p* = 0.0304) and LUSC (*p* < 0.001)—which means that higher expression of *TFAP2C* gene is characteristic for tumor cells in both subtypes. Furthermore, only in LUSC the *TFAP2C* expression had significantly negative correlation with infiltration of B cells (*p* < 0.001), T cells CD8+ (*p* < 0.001), neutrophils (*p* = 0.0015), and dendritic cells (*p* < 0.001). The results for *TFAP2D* (Figure 7D) showed that expression of this gene was positively correlated with tumor purity, but only in LUSC (*p* = 0.0267), which means that in LUSC the gene expression is connected to the tumor cells. Furthermore, *TFAP2D* mRNA level in LUSC was negatively correlated with T cell (CD8+) (*p* < 0.001) and neutrophil (*p* < 0.001) infiltration. For LUAD, *TFAP2D* was positively related to B cell (*p* < 0.001) and negatively related to three immune cells infiltration: T cell (CD8+) (*p* = 0.0010), macrophages (*p* = 0.0271) and neutrophils (*p* = 0.0456). For the *TFAP2E* gene (Figure 7E), the mRNA expression level was negatively correlated to CD8+ T cell abundance in both LUAD and LUSC (*p* = 0.003 and *p* < 0.001). The negative correlation was also observed to B cells, but only in LUSC subtype (*p* = 0.02). The positive correlation was observed between the CD4+ T cells infiltration level for LUAD and LUSC (*p* < 0.001 for both).

### 3.4. The Correlation between Survival and TFAP2 Family Members Expression in LC, LUAD and LUSC

**Figure 8:**
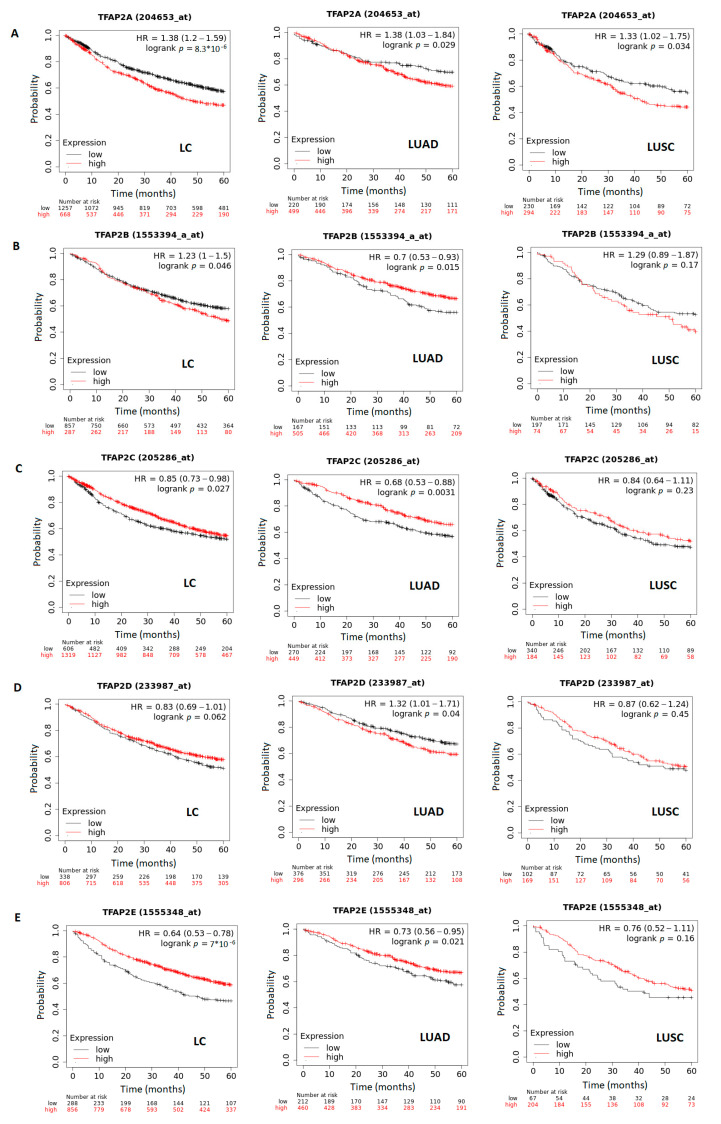
The correlation of *TFAP2* family genes expression (low vs. high expression level) with patient overall survival (OS) in lung cancer (LC), adenocarcinoma (LUAD), and squamous cell carcinoma (LUSC) for (**A**) *TFAP2A*, (**B**) *TFAP2B*, (**C**) *TFAP2C*, (**D**) *TFAP2D,* and (**E**) *TFAP2E*, respectively. The survival curves were retrieved from the Kaplan–Meier plotter (https://kmplot.com/analysis/, accessed on 20 November 2022) [32].

The correlation between patient survival and *TFAP2* genes family expression was analyzed by using KM plotter, with the follow-up threshold of 60 months (five years). The analysis evaluating the effect of the *TFAP2A* gene expression at the time of diagnosis on the survival time of NSCLC patients (Figure 8A) revealed that higher expression correlated with a shorter five years OS (*p* < 0.001) for overall lung cancer (LC) and after division to LUAD (*p* = 0.029) and LUSC (*p* = 0.034) subtypes. Next, for the *TFAP2B* gene, the survival curve analysis (Figure 8B) showed that the higher gene expression level is connected to shorter survival in lung cancer patients (*p* = 0.046); surprisingly, this finding is reversed for LUAD patients, for whom lower *TFAP2B* expression was associated with decreased patient survival rates (*p* = 0.015), and the significance is lost for LUSC subtype (*p* = 0.17). In the case of *TFAP2C* analysis (Figure 8C), the significant difference in survival according to mRNA expression level was found in LC (*p* = 0.027) and LUAD (*p* = 0.0031)—where a higher gene expression correlated with a longer OS—but not for the LUSC (*p* = 0.23) subtype. Analysis performed for *TFAP2D* gene (Figure 8D) revealed that there were no significant differences in the OS between the high- and low-expression group for LC patients (*p* = 0.062). This finding is also true for LUSC (*p* = 0.45), but not for the LUAD subtype, where there was a correlation between high *TFAP2D* gene expression level and shorter survival time (*p* = 0.04). Furthermore, poorer OS was significantly connected to a low *TFAP2E* gene expression level for LC (*p* < 0.001) and LUAD (*p* = 0.021), but not for LUSC (*p* = 0.16) patients (Figure 8E). Overall, it may seem that differential expression of this gene family is connected to patients’ survival rates, especially in the LUAD subtype, whereas it is generally not prognostic for LUSC patients, apart from the *TFAP2A* gene.

### 3.5. TFAP2 Family Members Alterations Frequency in LUAD and LUSC Patients

**Figure 9:**
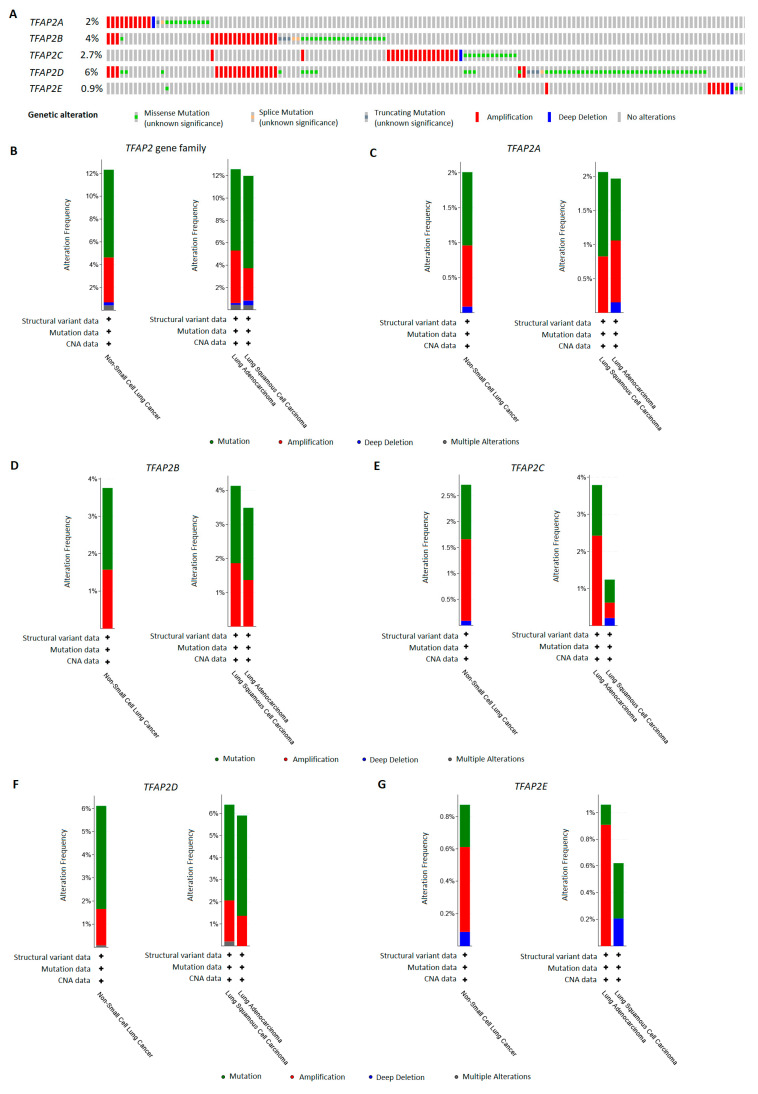
The alteration frequency of *TFAP2* family members in non-small cell lung cancer (NSCLC), lung adenocarcinoma (LUAD), and lung squamous cell carcinoma (LUSC) patients with cBioPortal (http://www.cbioportal.org/, accessed on 20 November 2022) [33,34]. Data presented for overall genetic alterations among *TFAP2* members (**A**), and with division into NSCLC, LUAD and LUSC for the *TFAP2* family (**B**), *TFAP2A* (**C**), *TFAP2B* (**D**), *TFAP2C* (**E**), *TFAP2D* (**F**), and *TFAP2E* (**G**).

The frequency of the *TFAP2* gene family alterations was evaluated by using the cBioPortal, using the data from TCGA database. Out of 1144 patients taken into analysis, queried genes were altered in 141 individuals. As shown in Figure 9A,B, this accounts for more than 12% of NSCLC patients, which exhibited modifications in the five *TFAP2* family genes, including mutations (7.7%), amplifications (3.9%), deep deletions (0.3%), and multiple alterations (0.4%). When analyzing individual alteration frequency, each *TFAP2* family member had rather low mutation rate (from 0.9% to 6%). After analyzing individual members, the *TFAP2A* gene was altered in 2% of all NSCLC patients, in LUAD modifications in this gene sequence constitute 2% of cases, the most common of which are mutations and amplifications. In LUSC, this gene is also altered in 2% of cases, including mutations, amplifications and deep deletions (Figure 9C). The alterations of *TFAP2B* gene were found in 4% of all NSCLC cases, and in 3.5% and 4.1% when divided into LUAD and LUSC subtypes, respectively (Figure 9D). Similarly, the most common modifications are mutations and amplifications. Almost 3% of NSCLC patients exhibited the alterations in *TFAP2C* gene, of which 3.8% accounted for LUAD and 1.2% for LUSC subtypes (Figure 9E). The observed alterations once again included missense mutations, amplifications in LUAD and LUSC, and deep deletions in LUSC. *TFAP2D* was the most frequently altered gene in NSCLC patients (6%), with similar frequency among LUAD (5.9%) and LUSC (6.4%) subtypes. The observed changes included mutations, amplifications in LUAD and LUSC and also multiple alterations in LUSC (Figure 9F). Finally, the *TFAP2E* gene was the least altered in the NSCLC patients (0.9%); the observed changes included mostly mutations and amplification in LUAD (1%), as well as mutations and deletions in LUSC (0.6%) cases (Figure 9G). Overall, the mutation frequency among all members of the *TFAP2* family is low, and the observed high gene expression level in lung cancer must result from other activating factors.

## 4. Discussion

The role of the *TFAP2* gene family in carcinogenesis is ambiguous. It was shown that the members of this family are involved in proliferation, apoptosis, angiogenesis, cell growth and that all these processes are characteristic for cancer. In addition, the target genes of these transcription factors are connected to the changes observed in many neoplasms. Some reports suggest that AP-2 factors can act bidirectionally and exert different functions depending on signaling pathway and type of the disease [12,14].

So far, scientific reports have focused mainly on specific *TFAP2* family members in the context of the development and progression of lung cancer and/or its individual subtypes. The most recent studies describe the first three genes encoding transcription factors, whereas less is known about *TFAP2D* and *TFAP2E*. Currently, there is no research available, that would comprehensively collect and describe information about all genes from the family and would differentiate the most common subtypes of LC, which is why this analysis was performed. From the above results, it is clear that the *TFAP2* family members are connected to the lung cancer occurrence, progression and survival and the difference is also observed between LUAD and LUSC patients.

The observed overexpression in most cancer types, including LUAD and LUSC, may suggest rather oncogenic potential of these gene family members, and this finding is most highlighted in the case of *TFAP2A* and *TFAP2C* genes through both TIMER and GEPIA databases. Similar results were obtained by Liao and Lin in the case of the *TFAP2A* gene in LUAD [35] and Cheng et al. [36], who reported higher *TFAP2A* and *TFAP2C* transcript levels in LUAD and LUSC patients as compared to normal tissue samples. Conversely in the case of *TFAP2B* gene, Cheng et al. reported no significant differences. On the other hand, a team lead by Fu et al. [37] found that *TFAP2B* was overexpressed in lung adenocarcinoma, which is consistent with the present analysis performed with the TIMER, but not with the GEPIA database. Previous studies of *TFAP2C* gene performed by Kang et al. [26] and Kim et al. [27] have shown overexpression in lung cancer cases, similar to this analysis. The reports for *TFAP2D* gene are scarce, but the latest research performed by Kołat et al. [38] indicated that high expression of this gene is observed in LC, with the highest values in LUAD, which is also true for current analysis performed on the TIMER clinical database. The *TFAP2E* gene seem to be the least studied of the family and no original reports concerning this gene expression level in lung cancer patients were found; nonetheless, through our analysis overexpression was found in LUAD, but not in LUSC patients in the TIMER, but not the GEPIA database. Given the above information, one should consider whether all members of the *TFAP2* family ought to be tested comprehensively in cases of lung cancer, due to their general upregulated nature of expression. The data about protein expression is mostly unavailable or incomplete through the accessed databases and the correlation between the genes to protein data could not be performed. Further studies evaluating the protein levels are needed to fully understand the role of these TF in lung cancer.

The association between clinicopathologic features, immune infiltration and changes in the expression level of *TFAP2* family genes is evident; in particular staging, *TP53* status, and smoking habits are the significant factors mentioned most frequently for all five genes. Cheng et al. [36] in their analysis found no associations for age, histology type, tumor stage and *TFAP2A*, *TFAP2B,* and *TFAP2C* gene expression level. This is moderately consistent with our analysis, where no correlations were found between all five gene family members and age and stage of LUAD patients. On the other hand, through analysis in LUSC patients, the significant differences were observed in *TFAP2B* transcript level according to tumor stage and age of patients. Unfortunately, Cheng et al. did not evaluate the smoking habits and *TP53* status. Several studies [17,39,40] have reported the mutual regulation between AP-2 factors and P53 protein. Li et al. [17] found that in human breast carcinoma cells the AP-2α and AP-2γ are transcriptionally regulated by P53, and the interaction is complex and involves promoters remodeling. Furthermore, Stabach et al. [39] reported that AP-2α also can regulate P53 transcriptional activity through coactivation and reduced stability. Results of this analysis suggest rather complex interaction between *TFAP2* genes expression level and *TP53* status of lung cancer patients, which stays in line with the previous reports. It was generally observed that higher *TFAP2A*, *TFAP2C,* and *TFAP2E* levels were observed in mutant *TP53* LUAD patients; on the other hand in case of LUSC subtype the significance was maintained only for *TFAP2C* gene. The reciprocal interaction between *TFAP2* family members and *TP53* seems to rely on the maintenance of the appropriate level of expression. Because mutations in *TP53* suggest the loss of its function, this in turn may translate into *TFAP2A*, *TFAP2C,* and *TFAP2E* getting out of transcriptional control and in result—their upregulation. This particular connection between the *TP53* and *TFAP2* TFs could be the starting point of novel, original research, which could help to elucidate the mutual interaction. It must be mentioned that in our analysis, the patients were divided into LUAD and LUSC subtypes, and we incorporated data from different databases and chosen diverse clinical factors, and this might be the reason of observed discrepancies.

To the best of our knowledge, this is the first study to investigate the expression level of *TFAP2* genes and tumor-infiltrating immune cells. There is an evident infiltration of diverse types of immune cells, including T cells, B cells, macrophages, natural killer (NK) cells, and dendritic cells, in NSCLC. A distinctive tumor microenvironment is created by immune cells with various functions and interactions, and differs in the case of LUAD and LUSC patients [41]. In the NSCLC microenvironment the T cells predominate, followed by B cells, macrophages, dendritic cells, and NK cells as the main immune cell subtypes [42]. The presence of dendritic cells is essential to activate the anticancer T lymphocytes to express particular antigens [43]. Impaired tumor infiltration can severely disturb the antitumor immune response of the body, which further affects the NSCLC development and progression, and in consequence patient survival [44]. Although in this study most of the correlations, apart from being statistically significant, were rather negligible and weak, it appears that the interaction between *TFAP2* gene family members and immune cells is complex and certainly related and differs between the two most common subtypes of lung cancer. However, to draw final conclusions more original studies are needed.

Differential expression of *TFAP2* gene family also influences the patients OS, it can be mentioned that *TFAP2A* may serve as a potential prognostic factor in both LUAD and LUSC, where high levels denoted higher mortality among patients. Similar results were reported by Liao and Lin [35] in LUAD and Cheng et al. [36] in overall lung cancer. No other associations with LUSC subtypes were found through this analysis. On the contrary, several more correlations with adenocarcinoma patients were exhibited. Surprisingly, the high expression level of *TFAP2B*, *TFAP2C,* and *TFAP2E* denoted longer five-year OS of LUAD patients through the Kaplan–Meier plotter (mean hazard ratio for all ~0.7), which may indicate the potentially protective nature of these members. The contrary results were obtained by Fu et al. [37] in the case of *TFAP2B* gene, where a higher transcript level was correlated with poorer OS in adenocarcinoma patients. On the other hand, Wang et al. [45] in their commentary indicated that the *TFAP2B* role in lung cancer is controversial and can be bidirectional. The finding for *TFAP2C* gene is in line with previous research by Chang et al. [21], who reported high gene expression level as an independent predictor and associated with a significantly better survival rate in LUAD. Moreover, Cheng et al. [36] reported no significant differences in OS of LC patients connected to *TFAP2C* gene expression level. In case of *TFAP2D,* gene current analysis revealed that the high expression level was connected to poor OS in LUAD patients, and this is in line with previous research by Kołat et al. [38]. Taken together, these findings indicate that there is a high probability that the *TFAP2* gene family may serve as useful prognostic biomarkers in LUAD, whereas these gene family members are not connected with LUSC patients’ survival. The observed discrepancies may result from the different number of samples taken for analysis and the difference in statistical methods from individual databases, and also for the end-time in preparing the survival curves, which was five years (60 months) in case of the current bioinformatics analysis.

Similar to recent article by Kołat et al. [38], through this analysis the *TFAP2D* gene was most altered from all family members, with a frequency of 6% in NSCLC. As the mutation frequency in all members of the family is rather low, the observed changes in gene expression must result also from other factors. Nevertheless, the recent report has shown that *TFAP2D* mutational status may depend on changes in the expression of other cancer-specific genes and targets of FDA-approved drugs, which has been confirmed in data from LUAD patients [38]. As this is a single report of a bioinformatics nature, research in this aspect must be continued to obtain proper validation and confirmation.

The present study revealed the gene expression level patterns, distinct prognostic values, and immune cell infiltration of five members of *TFAP2* family genes in LUAD and LUSC patients. Our research relied on public databases and was based on bioinformatics analyses. The obtained results must be interpreted with caution and several limitations must therefore be mentioned. First, some results and conclusions lack experimental verification and clinical validation. Secondly, the differences in databases, statistical methods, and potential sample heterogeneity may have caused the discrepancy between the results. Further verifications based on new original comprehensive studies with larger cohorts are required.

Nonetheless, the *TFAP2* genes family seems to play an important role in lung cancer pathogenesis, especially the *TFAP2A*, *TFAP2C,* and *TFAP2D* members in LUAD patients. Our bioinformatics research shows the new possible ways of targeting the differences in LUAD and LUSC subtypes and may become a starting point for new original clinical investigations, as there is still much to uncover about this gene family in lung cancer.

## Data Availability

The datasets used in this study are available publicly, links to the archives: https://cistrome.shinyapps.io/timer/ (accessed on 10 November 2022), http://gepia.cancer-pku.cn/index.html (accessed on 14 November 2022) http://ualcan.path.uab.edu/index.html (accessed on 16 November 2022), https://kmplot.com/analysis/ (accessed on 20 November 2022), http://www.cbioportal.org/ (accessed on 20 November 2022). All details are described in Section 2.
